# Assessment and Molecular Actions of Endocrine-Disrupting Chemicals That Interfere with Estrogen Receptor Pathways

**DOI:** 10.1155/2013/501851

**Published:** 2013-05-02

**Authors:** Gwenneg Kerdivel, Denis Habauzit, Farzad Pakdel

**Affiliations:** Institut de Recherche en Santé Environnement Travail (IRSET), INSERM U1085, TREC Team, SFR Biosit, University of Rennes 1, 35042 Rennes Cedex, France

## Abstract

In all vertebrate species, estrogens play a crucial role in the development, growth, and function of reproductive and nonreproductive tissues. A large number of natural or synthetic chemicals present in the environment and diet can interfere with estrogen signaling; these chemicals are called endocrine disrupting chemicals (EDCs) or xenoestrogens. Some of these compounds have been shown to induce adverse effects on human and animal health, and some compounds are suspected to contribute to diverse disease development. Because xenoestrogens have varying sources and structures and could act in additive or synergistic effects when combined, they have multiple mechanisms of action. Consequently, an important panel of *in vivo* and *in vitro* bioassays and chemical analytical tools was used to screen, evaluate, and characterize the potential impacts of these compounds on humans and animals. In this paper, we discuss different molecular actions of some of the major xenoestrogens found in food or the environment, and we summarize the current models used to evaluate environmental estrogens.

## 1. Introduction

**Figure 1:**
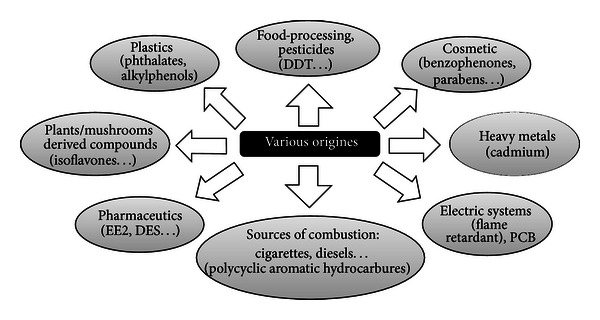
Sources of exposure to xenoestrogens. Various natural or synthetic molecules that enter in contact with humans by alimentation or during professional activities can interfere with estrogenic signaling pathways, explaining the great diversity of origins of the so-called xenoestrogens, illustrated here.

 Xenoestrogens are natural or industrial compounds found in the diet and environment that are capable of mimicking part of the effects of endogenous estrogens or interfering with estrogen signaling pathways [1]. Xenoestrogens are considered endocrine disruptors, also called endocrine disrupting chemicals (EDCs). The notion of endocrine disruptors appeared at the end of the 20th century, and these chemicals were defined as exogenous compounds that interfere with the signaling pathways of endogenous hormones at the level of their synthesis, storage, metabolism, transport, elimination, and binding to their specific receptors [2]. Additionally, EDCs are characterized by their ability to have deleterious effects on the health of living organisms and their descendants. EDCs can have numerous origins, various chemical structures, and act on various targets at the molecular level (Figure 1 and Table 1, see also [3, 4]).

Xenoestrogens, such as phthalates, can be extremely persistent in the environment. Some EDCs, for example, polychlorinated biphenyls (PCBs), are able to bioaccumulate in the food chain or in several biological matrices (as fats) and often exhibit effects at weak concentrations or in combination [5]. Among the numerous sources of exposure, the ingestion of water or contaminated food, cosmetics, pharmaceuticals, industrial exposure, and contact via professional activities (e.g., pesticides) are the most common. It is important to underline that the exposure to these compounds can have particularly critical effects at the fetal and postnatal stages [6]. Indeed, the development of the nervous system and the reproductive organs can be severely disrupted at these stages, as numerous tissues are particularly sensitive to hormonal regulation.

 Many xenoestrogens are synthetic estrogens stemming from human activity, which, due to their use, can enter in contact with living organisms or be released into the environment. For instance, workers in the production of contraceptive pills were exposed to the potent estrogen ethinyl-estradiol (EE2) which is capable of being absorbed by the skin [7]. A correlation was also established between the massive exposure to pesticide DDT (dichlorodiphenyl-trichloroethane) by farm laborers and the risks of oligospermia [8]. There is also the notorious example of diethylstilbestrol (DES), considered at its discovery as a miracle pill to fight against miscarriages and widely prescribed to pregnant women in the 70's in France. Exposure to this chemical *in utero* induced serious deformities and disorders in the reproductive system of male and female children [9]. More recently, an epidemiological study performed in the French West Indies analyzed the relationship between exposure to chlordecone and the risk of prostate cancer [10]. Chlordecone is an insecticide which exhibits estrogenic-like activity, that was used extensively (from 1973 to 1993) to control the banana root borer, thus contaminating the foodstuffs and population for several years. Interestingly, this study showed a significant increase in the risk of prostate cancer as the plasma chlordecone concentration increased [10]. Of course, these molecules are only a few of the numerous molecules whom estrogenic activities have been demonstrated or are suspected.

 As above mentioned, xenoestrogens are not only synthetic compounds, but there are also numerous natural molecules in food that exhibit estrogen-mimetic activities. These natural molecules are mainly phytoestrogens isoflavones, and the most consumed are genistein and daidzein; in particular, these two xenoestrogens are contained in the subproducts of soy and some legumes, fruits, and nuts. Other groups of phytoestrogens such as flavones, coumestans, and lignans were also found [11]. Additionally, certain mushrooms, mosses, or fungi can contain estrogen-like compounds called mycoestrogens, such as zearalenone [11].

## 2. Estrogen Receptor Pathways

**Figure 2:**
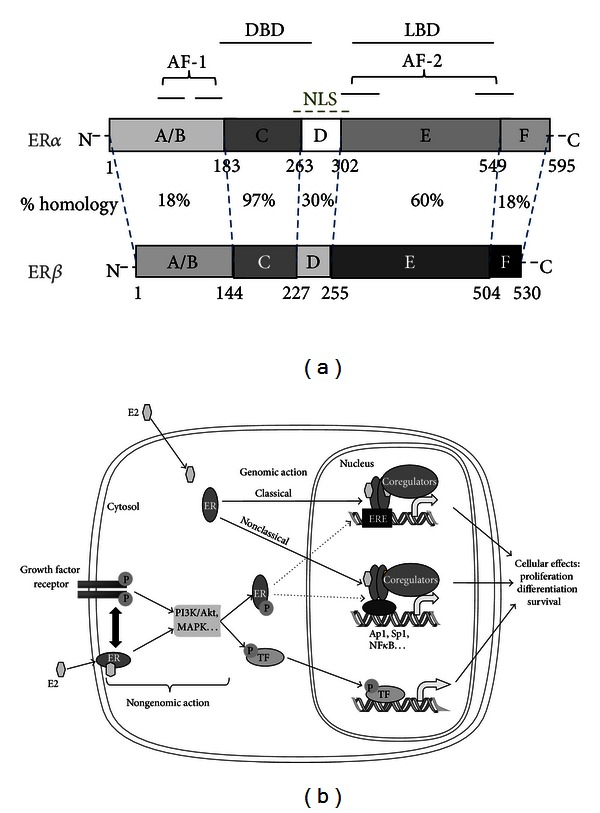
Structure and mechanisms of action of estrogen receptors. (a) ER*α* and ER*β* have an evolutionary conserved modular structure. The percentages of homology between the two forms are presented. The localizations of the ligand-binding domain (LBD) within the E domain and the DNA-binding domain (DBD) within the C domain are also presented. ERs possess two transactivation functions (AF-1 and AF-2), each divided into two subdomains which regulate the expression of target genes and contain a nuclear localization signal (NLS). (b) Due to its lipophilic properties, estradiol (E2) can passively enter the cell, through the lipid membranes. E2 can then bind ERs in the cytoplasm or the nucleus. ER dimers bind to the chromatin to modulate target gene expression. This mechanism corresponds to the genomic action of ERs, but ERs can also exercise nongenomic action, fast, directly in the cytoplasm. Indeed, the cytoplasmic or membrane-bound fraction of ER can induce, after E2-binding, the activation of intracellular signaling pathways independently or in association with the growth factor pathways.

 The physicochemical characteristics of estrogens, in particular their liposolubility, allow them to passively enter the cell through the plasma membrane. The majority of estrogen effects are mediated by their binding, in the cytoplasm or directly in nucleus, to estrogen receptors (ERs) which are expressed in numerous cell types. Two ERs, ER*α* (ESR1, NR3A1), and ER*β* (ESR2, NR3A2) were identified in mammals, although numerous splice variants exist (Figure 2(a)).

### 2.1. Characteristics of ERs

 ERs are members of the nuclear receptor superfamily which also includes the glucocorticoid receptor (GR), progesterone receptor (PGR), and androgen receptor (AR). The ability to act as a transcription factor whose activity depends on ligand binding is a common characteristic of most nuclear receptors. ERs are modular proteins that consist of distinct structural and functional domains. The A/B domain contains the ligand-independent transactivation function AF-1. The C domain contains the conserved zinc finger DNA-binding domain (DBD). The D domain contains nuclear localization signals (NLSs), and, finally, the E/F domain carries the ligand-dependent transactivation function AF-2 and the ligand-binding domain (LBD) [12, 13]. Mostly, the estrogen effects mediated by ER occur at the transcriptional level of a large number of estrogen-dependent genes [14–16]. These effects are called “genomic” actions as opposed to the nongenomic actions of estrogens that involve cytoplasmic signaling pathways (Figure 2(b)). These nongenomic effects are rapid effects of estrogens, of the order of a second or of a minute, which result in the activation of several intracellular signaling pathways such as MAPK or PI3K [17]. In addition, numerous studies have described the cross-talk between the genomic and nongenomic actions of ER, allowing a fine regulation of several target genes and increasing the complexity of the estrogenic signalization [18, 19] 

### 2.2. Mechanisms of ER Actions

 E2 mediates multiple phenotypic changes in cells by binding to its receptors, ERs that mediate E2 effects through diverse transcriptional mechanisms. Indeed, ERs modulate the expression of E2-target genes by directly binding to the chromatin at a consensus DNA sequence, the estrogen response element (ERE), within the promoter of target genes. This ER-DNA interaction induces the mobilization of the coregulators necessary for transcription (Figure 2(b)). This represents the classical pathway, but numerous E2-sensitive genes do not contain the ERE. ERs thus regulate transcription by interacting with other transcription factors, such as stimulating protein 1 (Sp1) or activator protein 1 (AP1), which are already bound to the promoter [20]. Ligand binding to the receptor induces ER conformational changes. The precise positioning of the helix H12, dependent on the nature of the bound-ligand, is essential for the interaction with coregulators and transcriptional activity of the ER. Thus, the expression of ER-target genes and ER-mediated cellular functions is dependent on both the promoter context and the nature of the estrogenic ligands [21, 22]. 

### 2.3. Tissue-Specific ER Expression

 ERs are coded by two different genes localized on two different chromosomes, chromosome 6 in the locus 6q25.1 for ER*α* and chromosome 14 in the connection between loci 14q11.1 and 14q11.2 for ER*β* [23, 24]. The utilization of different promoters results in multiple variants that code for the same protein, 66 kDa for ER*α* and 55 kDa for ER*β*, but the use of various promoters allows a fine tissue-specific regulation of ER expression, allowing for the modulation of transcript synthesis and regulating their stability and translational efficiency [25, 26]. In addition, several splice variants were identified. Among them, ER*α*-46 and the ER*α*-36 are the best-characterized forms. Due to the use of an alternative promoter resulting in the direct splicing to exon 2 of the ER*α* transcript, ER*α*-46 is deleted from the N-terminal part of the protein and lacks the AF-1 function. The ER*α*-46 isoform can inhibit the transcriptional activity of ER*α*-66 in various cell types [27, 28]. ER*α*-36 was discovered more recently and lacks both the N- and C-terminal domains, resulting in a form that lacks the two transactivation functions [29]. ER*α*-36 is capable of acting as a dominant negative form of ER*α*-66 and is also found anchored at the plasma membrane where it can modulate the activation of intracellular signaling pathways, such as the PI3K/Akt or MAPK signaling pathways.

 As a result, various tissues that express the ERs present very variable expression profiles of both ER subtypes. Thus, a strong expression of ER*α* is observed in tissues related to female reproduction (ovary, womb, mammary gland); ER*α* is also strongly expressed in men and is the most expressed ER subtype in the testicle (Leydig cells). ER*β* is also abundantly expressed in ovaries but expressed a little in the mammary gland. In men, ER*β* is expressed in the prostate, germinal cells, and epididymis. In both sexes, lung, hepatic, fat, osseous, nervous tissues, and endothelial cells express both receptors with variable expression levels [30, 31].

### 2.4. ER Expression during Development

 In addition to the diverse roles of estrogens in different target tissues, they have also multiple functions during development, particularly during the development of reproductive tissues such as the ovaries, uterus, and gonads. Estrogens play roles in the development of the brain, as they contribute to neuronal growth and differentiation [32]. ER knockout in mice demonstrated key roles for both ER*α* and ER*β* in gametogenesis. Interestingly, ER*α*-deficient mice exhibit significantly elevated levels of testicular testosterone secretion compared with wild-type fetal mice [32, 33]. The appearance of ERs appears to be under a spatial-temporally control during development [32]. For instance, the expression of ER*α* has been detected in the developing uterus as early as fetal day 15 in mesenchymal cells, whereas it appears in the epithelial cells at later fetal stages and increases during the neonatal period. In the rodent cerebral cortex, the expression of ER*α* is higher in postnatal life and decreases considerably during puberty [34]. However, ER*β* distribution in the developing brain of mice showed that ER*β* appears mainly in the midbrain and hypothalamus at E12.5, and its expression increased at E15.5 and E16.5. Interestingly, the expression of ER*β* appears strongly and widely throughout the brain including the cerebellum and striatum at E18.5, while very few positive cells could be detected in the ventricular region [35].

## 3. Mechanisms of Xenoestrogen Actions

**Figure 3:**
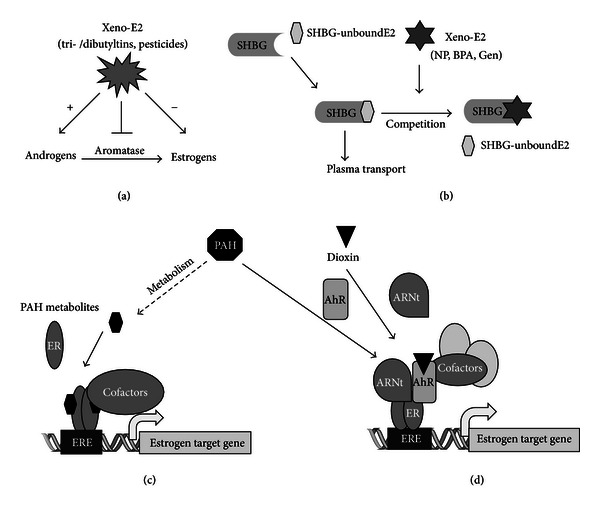
Examples of interaction between xenoestrogens and estrogen signaling pathways. (a) Some xenoestrogens, such as tributyltins, can inhibit aromatase, the enzyme responsible for the conversion of androgens in estrogens, resulting in the perturbation of the androgen/estrogen balance. (b) Other groups of compounds can interfere with estrogenic signaling by competing with natural estrogens for binding to sex hormone binding globulin (SHBG), resulting in defects in E2 plasma transport. ((c) and (d)) Interaction between polycyclic aromatic hydrocarbons (PAHs) and ERE-dependent E2-target gene transcription. (c) Some PAH metabolites can bind ER, resulting in the recruitment of ERs at the ERE and, subsequently, in the recruitment of coregulators that modulate the expression of E2 target genes. (d) Some PAH metabolites or dioxin are also capable of binding to the aryl hydrocarbon receptor (AhR), resulting in heterodimerization with aryl hydrocarbon nuclear translocator (Arnt). This transcriptionally active complex can then interact with ligand-unbound ER at the ERE site and modulate E2 target gene expression.

 Xenoestrogens can affect the endocrine system at every level. First, they can disrupt the action of the enzymes involved in steroidogenesis. For example, a perturbation of aromatase activity can modify the estrogen/androgen balance and thus alter the development or the function of reproductive organs, as was observed with the tributyltin and some other pesticides [36]. Other enzymes of the steroidogenesis can be impacted (mostly inhibited), as can the enzymes involved in metabolism of estrogens (Figure 3(a)). For instance, some PCB metabolites inhibit sulfotransferase, resulting in an increase of circulating estradiol rates [37]. The transport of hormones can also be used as the target of certain compounds capable of interacting with the binding sites of SHBG (sex hormone binding globulin), thus competing with endogenous estrogens (Figure 3(b)) [38].

 The most studied mode of actions of xenoestrogens is focusing the ability of these chemicals to bind and activate the ERs in target tissues [21]. However, it is of note that the two ERs mediate distinct biological effects in many tissues such as the mammary glands, bone, brain, and vascular system in both males and females. Therefore, because ER*α* and ER*β* show partially different tissue distribution and distinct physiological functions, xenoestrogens could display agonist or antagonist activity in a tissue-selective manner or during development. Considering the significant differences between ER subtypes in structural features and relative ligand binding affinity, xenoestrogens can induce distinct conformational changes in the tertiary structure of the ERs, affecting the recruitment of cofactors differently. These interactions between ERs and coactivators/corepressors are critical steps in ER-mediated transcriptional regulation and consequently the modulation of the expression of ER-target genes. For example, the phytoestrogen genistein exhibits an affinity for ER*β* that is 20-fold superior to its affinity for ER*α* [39]. Moreover, the genistein effect is often tissue specific because it depends on numerous factors such as the expression of specific cofactors, the ER*α*/ER*β* ratio, and the level of expression of certain intracellular kinases, including cytoplasmic tyrosine kinases. Genistein has been reported to have proliferative and antiproliferative effects in cancer cells [40].

 Xenoestrogens generally act in 100–1000 folds greater concentrations than estradiol but can have additive or synergic effects with endogenous estradiol or when they are present in combination [5]. Furthermore, the ability of some xenoestrogens to act as agonists in certain tissues and as antagonists in the others leads to the development and use of selective ER modulators (SERMs), in particular for antihormonal treatments, such as tamoxifen and raloxifene.

 Some xenoestrogens can also affect the ER nongenomic pathways and induce an endocrine disruption [41]. For instance, a recent study performed on structurally different xenoestrogens showed that at high concentrations, bisphenol A (BPA) and diethylstilbestrol (DES) are able to activate ERs via the activation of MAPK and PI3K in breast cancer cells. In addition, the activation of PKC by some xenoestrogens has been observed [42, 43]. Interestingly, PKC has been reported to modulate ER*α* transcriptional activity [44]. Therefore, synergic or additive effects between these pathways to combine the activation of ER signaling could be envisaged.

 Although the mechanistic studies on the interaction between dioxin and estrogen produced conflicting results, several studies reported that the ligands of AhR, such as polycyclic aromatic hydrocarbons (PAHs), can also affect estrogenic signaling in mammary or uterine cells (Figure 3(c)). Ohtake et al. [45] showed that an AhR agonist, methylcholanthrene (3MC), is able to activate a reporter gene containing an ERE without affecting the expression level of ER. However, when this promoter is activated by estradiol, 3MC has an antagonistic effect. Coimmunoprecipitation assays showed that these functional interactions are correlated with the physical interaction between AhR and ER. The proposed model (Figure 3(d)) suggests that 3MC activates AhR, which dimerizes with Arnt. The AhR/ARNT heterodimer can directly associate with ER that is not ligand bound to activate estrogen-sensitive gene transcription by recruiting the coactivator p300 [45]. This model is consolidated by *in vivo* experiments performed in the mouse. In fact, the proliferative effect of 3MC on the uterus is observed in ovariectomized mice, but not in AhR knockout mice. These studies suggest an original mechanism of activation of ER*α* in the absence of estradiol because ligand-activated AhR is able to cooperate with the ER that is not ligand bound to activate transcription. However, some metabolites of AhR ligands, including 3MC, could also behave as partial agonist on estrogen signaling pathways by direct interaction with ER*α* (Figure 3(a)). 

 Several mechanisms have also been proposed to describe the antiestrogenicity of AhR ligands [46]. By binding to AhR, these compounds could interfere with transcriptionally active ER/SP1 or ER/AP-1 complexes [47–49]. They can also inhibit the binding of ER to ERE sites by direct association with ER*α* [45]. The antiestrogenic effects of dioxins could also be mediated by the reduction of ER*α* protein level through activation of the proteasome [50]. However, AhR-mediated degradation rates may vary according to the specific cellular context [46]. Therefore, these interactions should be taken into account in the interpretations of studies that investigate the estrogenic effects of AhR ligands, particularly in mixtures. 

 Similarly, both the potent estrogenic and antiestrogenic effects of the heavy metal cadmium (Cd) have been reported *in vitro* in mammary cell lines, recombinant yeast assays, or fish hepatocyte cultures and *in vivo *in the rodent uterus [51–53]. Although the precise mechanisms underlying the effects of Cd as an endocrine disruptor remain unclear [54], two different mechanistic explanations were suggested. Cd could directly interact with the LBD of ER*α*, inducing a conformational change in LBD that favors the interaction between helix 12 of ER*α* with transcriptional coactivators [55]. Other studies suggested that the interaction of Cd with the LBD of ER*α* induces conformational changes in the DBD which could inactivate the DNA binding activity of the receptor, reducing transactivation [51, 53]. However, cadmium is not the sole heavy metal able to interfere with estrogen signaling pathways, even if the precise modes of actions of these metalloestrogens are largely misunderstood [56, 57].

 Because EDC can also modulate hormonal signaling indirectly via their metabolites, EDC metabolism should be taken into account in the evaluation and identification of their mechanisms of action. For instance, the insecticide DDT and its metabolite DDE (dichlorodiphenyldichloroethylene) were characterized as weak estrogens in the environment and were suspected to affect reproduction function in several animal species [58]. More recently, DDE, which is highly lipophilic and resistant to biodegradation, was identified as the compound that induced the feminization of alligators (i.e., micropenis and various abnormalities of the testes) from Lake Apopka. These effects are likely mediated by the inhibition of androgen signaling during the critical developmental window [59]. In fact, although DDE shows low affinity to ERs, it is capable of binding to AR and repressing the transcriptional activity of this receptor. This antiandrogenic action of DDE shown in different cell-based assays could clearly cause abnormalities in the steroidogenic cells of rat testis and could disturb the development and function of fetal testis [60–62]

## 4. Assessment and Quantification Methods, Biosensors, and Bioassays

 In environmental monitoring, there are two major questions: what is the quantity of each pollutant in an environmental sample, and what is the molecule's effect on humans and wildlife? To answer these two questions, several methods have been developed. These methods have progressed with the understanding of estrogenic functions at the organismal, organ, cell, and molecular levels. Due to this diversity of EDC actions, evaluation and quantification require physicochemical, biophysical, biochemical, cellular, and whole organism-based methods.

### 4.1. Analytical Methods

 The most widely used methods for the quantification of estrogenic compounds are analytical methods such as high-performance liquid chromatography (HPLC), gas chromatography/mass spectrometry (GC/MS), GC-spectrometry of mass coupled (MS/MS), and liquid-phase chromatography (LC-MS/MS). These methods allow the extensive identification and quantification of compounds with estrogenic properties, within solid or liquid samples [63]. However, these methods are not directly sensitive enough for the direct measurement of the estrogenic compounds contained in an environmental sample. These methods need therefore a preconcentration step to increase the concentration of compounds between 100 and 1000 folds. Moreover, the preconcentration step is aimed to specifically extract the estrogenic compound through liquid-liquid, solid-phase extraction (SPE), solid-phase microextraction (SPME) or stir bar sorptive extraction (SBSE) [64–69]. These extractions methods are determined by the chemical properties of the target molecules. The elution leads to specific concentration of the compound in the adequate solvent for chromatography analysis. For a technical review see Farré et al. [70]. Together, these techniques allow for a precise detection of the compounds with a low limit of detection, but they do not provide information about the estrogenic or antiestrogenic properties of these molecules. Furthermore, these methods target specific molecules which imply that the estrogenic potential of the molecules of interest was already identified. However, using bioassays ranging from *in vitro* receptor binding assays, tissue culture, and cell-based assays, and *in vivo* animal models can overcome most analytical drawbacks. 

### 4.2. *In Vivo* Methods for Estrogenic Potency Assessment

 The use of the whole organism methods presents the advantage of an *in vivo* evaluation of the estrogenic potential of molecules in biological functions or in the expression of markers of hormonal exposure. These approaches have been developed in amphibians, fishes, rats, and mice to estimate the estrogenicity of compounds [71]. The uterotrophic test is based on the strong proliferative effect that estrogens have in the rodent female genital tract. This test is commonly conducted with measurements of the uterus weight of immature or ovariectomized rodents. This test has widely been used by researchers for estrogenic compound evaluation [72, 73] and has been validated by OECD and the endocrine disrupters testing assessment group (EDTA) [74]. Other *in vivo* tests examine the expression of the vitellogenin of male fishes, by ELISA or Western blot [75]. The induction of vitellogenin after exposure to estrogenic compounds has been demonstrated in several fish species [76, 77]. Transgenic mice and zebrafish that express an easily quantifiable reporter gene were also recently developed. These models allow for the specific expression of estrogen-dependent genes in different cell types [78–81]. 

### 4.3. *In Vitro* Cell-Based Methods for Evaluation and Quantification

*In vitro* cell-based assays using cell lines offer a good sensibility but do not allow the determination of the specific effect of a particular xenoestrogen in an environmental sample containing several compounds. The use of these assays in environmental monitoring gives global information of the estrogenic potency of the sample. Moreover, these *in vitro* bioassays do not elucidate the overall effects of the biotransformation and pharmacokinetics of compounds. However, these assays provide a method to quickly estimate the total estrogenicity of a mixture or given compound and generally require less expensive equipment than the analytical methods. 

 Several bioassays have been developed for the estrogenic potency assessment. The estrogenic actions evaluated by these methodologies are based on the estrogenic action in cells, for instance, the proliferation of ER-positive breast cancer cell lines (MCF7, T47D), known as E-screen [82], and optimized by several authors to ameliorate detection [82–85]. Other bioassays are based on the capacity of estrogenic compounds to bind and activate ER. These assays that induce an estrogen-regulated gene were previously reported as ER-CALUX [86], YES assay in yeast [87–89] and various reporter gene assays [90, 91]. These assays target ERE, SP1, and AP1 regulated genes [22, 92]. Other methods use the differentiation of ER-positive cell lines to evaluate the estrogenic potency of EDC [93]. Together, these methods permit the evaluation of estrogenic potency of compounds and, in some cases when test is sensitive enough, the environmental quantification of estrogenic compounds [83, 85, 94].

### 4.4. Biosensor

The term biosensor appeared in 1962 when the first method was developed for the detection of glucose concentration in blood sample thanks to amperometric method [95]. A biosensor consists of two parts, the biological recognition element and the transducer. The biological recognition element is able to interact specifically with the target, while the transducer is able to convert the biological recognition event into an electrical signal because of physical property changes (Table 2). Several strategies have been established to optimize the couple biological transducer [96, 97].

 As detailed in Table 2, there are many possibilities in the combination of biological recognition element and transducer. However, in the development of methods for the evaluation and quantification of estrogenic compounds, the main biological recognition elements used are as follows: antibodies against estradiol [98], estrogen receptor (complete ER protein [99, 100], LBD [101, 102] or recombinant and genetically modified ER [103–105]), ER dimerization [106], DNA binding [107–111], and finally the ER interaction with cofactors. The transducers usually used are the following: fluorescence anisotropy [112–114], surface plasmon resonance (SPR) [108–111], reflectometric interference spectroscopy (RfIS) [102], fluorescence resonance energy transfer (FRET) [115], and bioluminescence resonance energy transfer (BRET) [103, 104]. However, the high diversity of the biological recognition elements and transducers that are usually used in biosensor methods for the evaluation of estrogenic compounds makes comparison between methods difficult. While the time of responsiveness from these methods is generally shorter than with the cellular methods, it is currently not sensitive enough to use them for environmental detection. Therefore, for environmental monitoring purposes, a pre-concentration step is currently needed.

## 5. Conclusion

 The origin and the exposure sources of xenoestrogens are multiple. They can come from food, products of combustion, and agricultural and industrial chemicals. Because xenoestrogens have varying structural complexity and produce a great number of metabolites or biodegradation products in the environment, they exhibit various mechanisms of action. Moreover, these mechanisms could differ depending on the cellular and tissue context. For instance, it has been reported that the widespread environmental contaminants PAHs have both estrogenic and antiestrogenic activity [116]. Similarly, both potent estrogenic and antiestrogenic effects and an androgen-like effect of Cd have been shown *in vivo* and *in vitro* [51–53]. 

 In addition to the direct actions of xenoestrogens in primarily exposed organisms which usually result in the modulation of gene expression and potentially in phenotype alterations, there is increasing evidence to suggest that EDC can also act across generations. For instance, a study in a mouse model showed an increase in uterine adenocarcinoma in the female descendants (lineage F2) of mice exposed developmentally to diethylstilbestrol [117]. Parental exposure to environmental contaminants could thus induce epigenetic modifications and gene expression alterations that can pass from one generation to the next, resulting in physiological changes in their offspring [118]. It has been reported that the exposure during development to the fungicide vinclozolin induces a reduction of fertility in treated male animals that is transmitted through four generations without further exposure to vinclozolin [119–121]. This pesticide has been characterized as an antiandrogenic compound, and some of its metabolites could interact with other steroid receptors including the receptors for progesterone, glucocorticoids, and mineralocorticoids. Thus, vinclozolin could interfere with hormone signaling pathways during development, but it is currently not known whether the effects of vinclozolin are mediated by its interference with hormonal signaling during development [122–125]. 

 These studies exemplify the diversity and complexity of xenoestrogen effects and the need for the further understanding of the diversity of their molecular actions. In particular, the effort concerning xenoestrogen effects in epigenetic modifications at the DNA sequences and chromatin-associated proteins should be a priority research.

 It is important to emphasize that xenoestrogens do not necessarily mediate their effects by binding to specific nuclear ERs. Indirect effects can thus be considered. For instance, modifications on the expression or activity of their associated protein kinases, enzymes, or transcription factors necessary for the activity of the specific ER subtypes (DNA binding, phosphorylation, transactivation, degradation, and subcellular translocation) [37, 40]. 

 However, identifying the G-protein-coupled receptor homologue GPR30 as the plasma membrane receptor for estrogens provides a higher level of complexity to the mechanisms of action of these hormones [126]. GPR30 is able to bind 17*β*-estradiol and allows fast nongenomic responses of estrogens such as the stimulation of MAPK pathways, adenylyl cyclase, or c-fos expression in the breast cancer cell line SKBR3 which does not express the classical ERs [127–129]. Notably, several phytoestrogens, such as genistein and quercetin, or other xenoestrogens such as bisphenol A, zearalenone, and nonylphenol, have been shown to bind to this membrane estrogen receptor [130, 131]. Because GPR30 is expressed in a wide number of cell types, it could potentially mimic environmental estrogen effects in a great number of tissues. The further characterization of cellular and tissue distribution and the mode of action of GPR30 and other plasma membrane receptors for steroid hormones will likely contribute to a better comprehension of the xenoestrogen actions in relation to the important number of physiological roles played by estrogens. 

 The assessment of environmental estrogens has greatly increased in the past decade in different areas such as the development of biomarkers, cell- and animal-based bioassays, bioinformatics, and bioanalytical and biosensor technology [51, 78, 82, 86, 110]. To elucidate the estrogenic or antiestrogenic properties of suspected compounds, several *in vivo* screening approaches, which generally cover the kinetics and potential degradation of compounds, were developed. A transgenic mouse model expressing an estrogen-dependent green Fluorescent Protein (GFP)-based reporter gene constitutes a powerful animal model because it provides a method to determine the *in vivo* delivery, stability, and tissue specificity of the compounds within the mammalian body [79]. More recently, a similar method was adapted to nonmammalian vertebrates such as zebrafish [78, 80, 132]. Transgenic zebrafishes constitute an interesting animal model because of their rapid and ex-utero development, the transparency of their embryos, and their small size. While the assessment of xenoestrogens by *in vitro* assays does not fully take into account metabolism and pharmacokinetics, some of these assays are notably valuable tools for (i) the high specificity of responsiveness, (ii) the high throughput screening of large numbers of chemicals, and (iii) the determination of molecular and cellular actions of the environmental contaminants and identification of their signalization pathways and cofactor and ER selectivity. Therefore, the combination of *in vivo* and *in vitro* approaches is necessary to obtain a better understanding of the molecular actions of xenoestrogens.

## Figures and Tables

**Table 1:** Illustration of the structural diversity of estrogenic compounds from diverse origins.

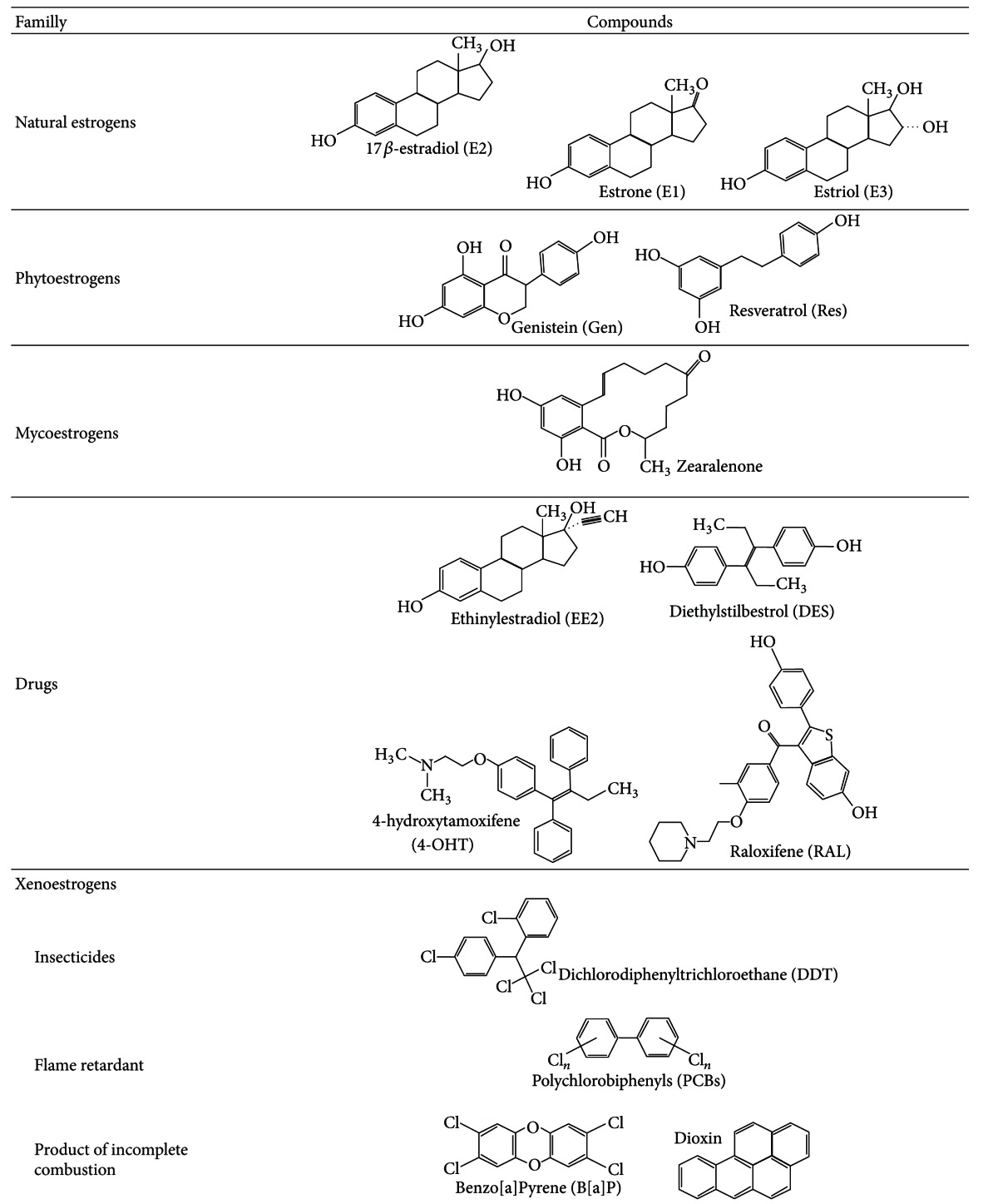 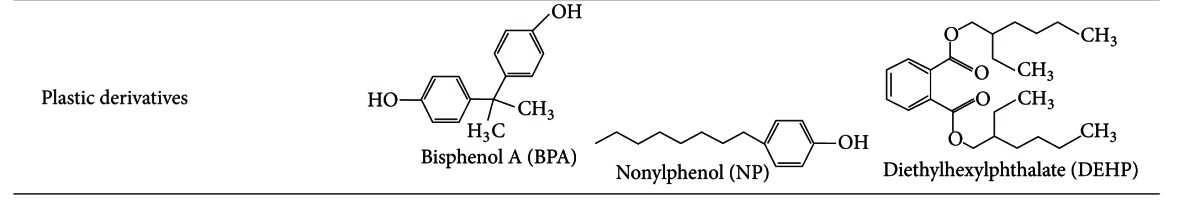

**Table 2:** Biological recognition elements and transducers usually used in the development of biosensors.

Biological recognition elements	Transducers
(i) Cells (ii) Membranes (iii) Nucleic acids (iv) Protein: (a) Antibodies (b) Enzymes (c) Membrane receptors (d) Nuclear receptors (e) Peptides	(i) Optical (a) Fluorescence (BRET, FRET, fluorescence anisotropy *⋯*) (b) Colorimetry (c) Surface plasmon resonance (SPR) (d) Reflectometric interference spectroscopy (RfIS) (ii) Electrochemical (a) Amperometric (b) Conductimetry (c) Potentiometry (iii) Piezoelectric (a) Quartz crystal microbalance (QCM) (iv) Thermal (a) Differential scanning microcalorimetry (DSC) (b) Isothermal microcalorimetry (ITC).

## References

[1] Pelekanou V, Leclercq G (2011). Recent insights into the effect of natural and environmental estrogens on mammary development and carcinogenesis. *The International Journal of Developmental Biology*.

[2] Kavlock RJ, Daston GP, DeRosa C (1996). Research needs for the risk assessment of health and environmental effects of endocrine disrupters: a report of the U.S. EPA-sponsored workshop. *Environmental Health Perspectives*.

[3] Shanle EK, Xu W (2011). Endocrine disrupting chemicals targeting estrogen receptor signaling: identification and mechanisms of action. *Chemical Research in Toxicology*.

[4] Lóránd T, Vigh E, Garai J (2010). Hormonal action of plant derived and anthropogenic non-steroidal estrogenic compounds: phytoestrogens and xenoestrogens. *Current Medicinal Chemistry*.

[5] Kortenkamp A, Altenburger R (1998). Synergisms with mixtures of xenoestrogens: a reevaluation using the method of isoboles. *Science of the Total Environment*.

[6] Singleton DW, Khan SA (2003). Xenoestrogen exposure and mechanisms of endocrine disruption. *Frontiers in Bioscience*.

[7] Harrington JM, Stein GF, Rivera RO, de Morales AV (1978). The occupational hazards of formulating oral contraceptives. A survey of plant employees. *Archives of Environmental Health*.

[8] Degen GH, Bolt HM (2000). Endocrine disruptors: update on xenoestrogens. *International Archives of Occupational and Environmental Health*.

[9] Newbold RR (2008). Prenatal exposure to diethylstilbestrol (DES). *Fertility and Sterility*.

[10] Multigner L, Ndong JR, Giusti A (2010). Chlordecone exposure and risk of prostate cancer. *Journal of Clinical Oncology*.

[11] Liu ZH, Kanjo Y, Mizutani S (2010). A review of phytoestrogens: their occurrence and fate in the environment. *Water Research*.

[12] Evans RM (1988). The steroid and thyroid hormone receptor superfamily. *Science*.

[13] Pike ACW (2006). Lessons learnt from structural studies of the oestrogen receptor. *Best Practice and Research*.

[14] Boudot A, Kerdivel G, Habauzit D (2011). Differential estrogen-regulation of CXCL12 chemokine receptors, CXCR4 and CXCR7, contributes to the growth effect of estrogens in breast cancer cells. *PLoS ONE*.

[15] Cicatiello L, Scafoglio C, Altucci L (2004). A genomic view of estrogen actions in human breast cancer cells by expression profiling of the hormone-responsive transcriptome. *Journal of Molecular Endocrinology*.

[16] Kerdivel G, Boudot A, Pakdel F (2013). Estrogen represses CXCR7 gene expression by inhibiting the recruitment of NF*κ*B transcription factor at the CXCR7 promoter in breast cancer cells. *Biochemical and Biophysical Research Communications*.

[17] Nilsson S, Mäkelä S, Treuter E (2001). Mechanisms of estrogen action. *Physiological Reviews*.

[18] La Rosa P, Pesiri V, Leclercq G, Marino M, Acconcia F (2012). Palmitoylation regulates 17*β*-estradiol-induced estrogen receptor-*α* degradation and transcriptional activity. *Molecular Endocrinology*.

[19] Pedram A, Razandi M, Aitkenhead M, Hughes CCW, Levin ER (2002). Integration of the non-genomic and genomic actions of estrogen: membrane-initiated signaling by steroid to transcription and cell biology. *The Journal of Biological Chemistry*.

[20] Safe S, Kim K (2008). Non-classical genomic estrogen receptor (ER)/specificity protein and ER/activating protein-1 signaling pathways. *Journal of Molecular Endocrinology*.

[21] Celik L, Lund JDD, Schiøtt B (2008). Exploring interactions of endocrine-disrupting compounds with different conformations of the human estrogen receptor *α* ligand binding domain: a molecular docking study. *Chemical Research in Toxicology*.

[22] Kerdivel G, Le Guevel R, Habauzit D, Brion F, Ait-Aissa S, Pakdel F (2013). Estrogenic potency of benzophenone UV filters in breast cancer cells: proliferative and transcriptional activity substantiated by docking analysis. *PLoS ONE*.

[23] Gosden JR, Middleton PG, Rout D (1986). Localization of the human oestrogen receptor gene to chromosome 6q24–q27 by in situ hybridization. *Cytogenetics and Cell Genetics*.

[24] Enmark E, Pelto-Huikko M, Grandien K (1997). Human estrogen receptor *β*-gene structure, chromosomal localization, and expression pattern. *Journal of Clinical Endocrinology and Metabolism*.

[25] Flouriot G, Griffin C, Kenealy M, Sonntag-Buck V, Gannon F (1998). Differentially expressed messenger RNA isoforms of the human estrogen receptor-*α* gene are generated by alternative splicing and promoter usage. *Molecular Endocrinology*.

[26] Okuda Y, Hirata S, Watanabe N, Shoda T, Kato J, Hoshi K (2003). Novel splicing events of untranslated first exons in human estrogen receptor alpha (ER*α*) gene. *Endocrine Journal*.

[27] Flouriot G, Brand H, Denger S (2000). Identification of a new isoform of the human estrogen receptor-alpha (hER-*α*) that is encoded by distinct transcripts and that is abide to repress hER-*α* activation function 1. *The EMBO Journal*.

[28] Penot G, Le Péron C, Mérot Y (2005). The human estrogen receptor-alpha isoform hERalpha46 antagonizes the proliferative influence of hERalpha66 in MCF7 breast cancer cells. *Endocrinology*.

[29] Zhao YW, Zhang X, Shen P, Loggie BW, Chang Y, Deuel TF (2005). Identification, cloning, and expression of human estrogen receptor-*α*36, a novel variant of human estrogen receptor-*α*66. *Biochemical and Biophysical Research Communications*.

[30] Shi H, Shigeta H, Yang N, Fu K, O’Brian G, Teng CT (1997). Human estrogen receptor-like 1 (ESRL1) gene: genomic organization, chromosomal localization, and promoter characterization. *Genomics*.

[31] Saunders PTK (1998). Oestrogen receptor beta (ER*β*). *Reviews of Reproduction*.

[32] Couse JF, Korach KS (1999). Estrogen receptor null mice: what have we learned and where will they lead us?. *Endocrine Reviews*.

[33] Delbès G, Levacher C, Duquenne C, Racine C, Pakarinen P, Habert R (2005). Endogenous estrogens inhibit mouse fetal leydig cell development via estrogen receptor *α*. *Endocrinology*.

[34] Wilson ME, Westberry JM, Trout AL (2011). Estrogen receptor-alpha gene expression in the cortex: sex differences during development and in adulthood. *Hormones and Behavior*.

[35] Fan X, Kim HJ, Warner M, Gustafsson JA (2007). Estrogen receptor *β* is essential for sprouting of nociceptive primary afferents and for morphogenesis and maintenance of the dorsal horn interneurons. *Proceedings of the National Academy of Sciences of the United States of America*.

[36] Heidrich DD, Steckelbroeck S, Klingmuller D (2001). Inhibition of human cytochrome P450 aromatase activity by butyltins. *Steroids*.

[37] Kester MHA, Bulduk S, Tibboel D (2000). Potent inhibition of estrogen sulfotransferase by hydroxylated PCB metabolites: a novel pathway explaining the estrogenic activity of PCB’s. *Endocrinology*.

[38] Déchaud H, Ravard C, Claustrat F, de la Perrière AB, Pugeat M (1999). Xenoestrogen interaction with human sex hormone-binding globulin (hSHBG). *Steroids*.

[39] Routledge EJ, White R, Parker MG, Sumpter JP (2000). Differential effects of xenoestrogens on coactivator recruitment by estrogen receptor (ER) *α* and ER*β*. *The Journal of Biological Chemistry*.

[40] Jagadeesh S, Kyo S, Banerjee PP (2006). Genistein represses telomerase activity via both transcriptional and posttranslational mechanisms in human prostate cancer cells. *Cancer Research*.

[41] Watson PH, Pon RT, Shiu RPC (1991). Inhibition of c-myc expression by phosphorothioate antisense oligonucleotide identifies a critical role for c-myc in the growth of human breast cancer. *Cancer Research*.

[42] Martin F, Reig JA, Soria B (1995). Secretagogue-induced [Ca^2+^]_i_ changes in single rat pancreatic islets and correlation with simultaneously measured insulin release. *Journal of Molecular Endocrinology*.

[43] Li X, Zhang S, Safe S (2006). Activation of kinase pathways in MCF-7 cells by 17*β*-estradiol and structurally diverse estrogenic compounds. *Journal of Steroid Biochemistry and Molecular Biology*.

[44] Cho H, Katzenellenbogen BS (1993). Synergistic activation of estrogen receptor-mediated transcription by estradiol and protein kinase activators. *Molecular Endocrinology*.

[45] Ohtake F, Takeyama KI, Matsumoto T (2003). Modulation of oestrogen receptor signalling by association with the activated dioxin receptor. *Nature*.

[46] Safe S, Wormke M (2003). Inhibitory aryl hydrocarbon receptor-estrogen receptor *α* cross-talk and mechanisms of action. *Chemical Research in Toxicology*.

[47] Khan S, Barhoumi R, Burghardt R, Liu S, Kim K, Safe S (2006). Molecular mechanism of inhibitory aryl hydrocarbon receptor—estrogen receptor/Sp1 cross talk in breast cancer cells. *Molecular Endocrinology*.

[48] Matthews J, Wihlén B, Thomsen J, Gustafsson JA (2005). Aryl hydrocarbon receptor-mediated transcription: ligand-dependent recruitment of estrogen receptor *α* to 2,3,7,8-tetrachlorodibenzo-p-dioxin- responsive promoters. *Molecular and Cellular Biology*.

[49] Pocar P, Fischer B, Klonisch T, Hombach-Klonisch S (2005). Molecular interactions of the aryl hydrocarbon receptor and its biological and toxicological relevance for reproduction. *Reproduction*.

[50] Wormke M, Stoner M, Saville B (2003). The aryl hydrocarbon receptor mediates degradation of estrogen receptor *α* through activation of proteasomes. *Molecular and Cellular Biology*.

[51] Le Guével R, Petit FG, Le Goff P, Métivier R, Valotaire Y, Pakdel F (2000). Inhibition of rainbow trout (*Oncorhynchus mykiss*) estrogen receptor activity by cadmium. *Biology of Reproduction*.

[52] Johnson MD, Kenney N, Stoica A (2003). Cadmium mimics the *in vivo* effects of estrogen in the uterus and mammary gland. *Nature Medicine*.

[53] Silva E, Lopez-Espinosa MJ, Molina-Molina JM, Fernández M, Olea N, Kortenkamp A (2006). Lack of activity of cadmium in *in vitro* estrogenicity assays. *Toxicology and Applied Pharmacology*.

[54] Takiguchi M, Yoshihara S (2006). New aspects of cadmium as endocrine disruptor. *Environmental Sciences*.

[55] Stoica A, Katzenellenbogen BS, Martin MB (2000). Activation of estrogen receptor-*α* by the heavy metal cadmium. *Molecular Endocrinology*.

[56] Byrne C, Divekar SD, Storchan GB, Parodi DA, Martin MB (2013). Metals and breast cancer. *Journal of Mammary Gland Biology and Neoplasia*.

[57] Divekar SD, Storchan GB, Sperle K (2011). The role of calcium in the activation of estrogen receptor-alpha. *Cancer Research*.

[58] Guillette LJ, Gross TS, Masson GR, Matter JM, Percival HF, Woodward AR (1994). Developmental abnormalities of the gonad and abnormal sex hormone concentrations in juvenile alligators from contaminated and control lakes in Florida. *Environmental Health Perspectives*.

[59] Kelce WR, Stone CR, Laws SC, Gray LE, Kemppainen JA, Wilson EM (1995). Persistent DDT metabolite p,p'-DDE is a potent androgen receptor antagonist. *Nature*.

[60] Yamasaki K, Okuda H, Takeuchi T, Minobe Y (2009). Effects of *in utero* through lactational exposure to dicyclohexyl phthalate and p,p'-DDE in Sprague-Dawley rats. *Toxicology Letters*.

[61] Thiel A, Guth S, Böhm S, Eisenbrand G (2011). Dicofol degradation to p,p'-dichlorobenzophenone—a potential antiandrogen. *Toxicology*.

[62] Adamsson A, Salonen V, Paranko J, Toppari J (2009). Effects of maternal exposure to di-isononylphthalate (DINP) and 1,1-dichloro-2,2-bis(p-chlorophenyl)ethylene (p,p'-DDE) on steroidogenesis in the fetal rat testis and adrenal gland. *Reproductive Toxicology*.

[63] Campbell CG, Borglin SE, Green FB, Grayson A, Wozei E, Stringfellow WT (2006). Biologically directed environmental monitoring, fate, and transport of estrogenic endocrine disrupting compounds in water: a review. *Chemosphere*.

[64] Rodriguez-Mozaz S, de Alda MJL, Barceló D (2005). Picogram per liter level determination of estrogens in natural waters and waterworks by a fully automated on-line solid-phase extraction-liquid chromatography-electrospray tandem mass spectrometry method. *Analytical Chemistry*.

[65] Peñalver A, Pocurull E, Borrull F, Marcé RM (2002). Method based on solid-phase microextraction-high-performance liquid chromatography with UV and electrochemical detection to determine estrogenic compounds in water samples. *Journal of Chromatography A*.

[66] Carpinteiro J, Quintana JB, Rodríguez I, Carro AM, Lorenzo RA, Cela R (2004). Applicability of solid-phase microextraction followed by on-fiber silylation for the determination of estrogens in water samples by gas chromatography-tandem mass spectrometry. *Journal of Chromatography A*.

[67] Bianchi F, Mattarozzi M, Careri M (2010). An SPME-GC-MS method using an octadecyl silica fibre for the determination of the potential angiogenesis modulators 17*β*-estradiol and 2-methoxyestradiol in culture media. *Analytical and Bioanalytical Chemistry*.

[68] Nogueira JMF (2012). Novel sorption-based methodologies for static microextraction analysis: a review on SBSE and related techniques. *Analytica Chimica Acta*.

[69] Mol HGJ, Sunarto S, Steijger OM (2000). Determination of endocrine disruptors in water after derivatization with N-methyl-N-(tert.-butyldimethyltrifluoroacetamide) using gas chromatography with mass spectrometric detection. *Journal of Chromatography A*.

[70] Farré M, Kantiani L, Petrovic M, Pérez S, Barceló D (2012). Achievements and future trends in the analysis of emerging organic contaminants in environmental samples by mass spectrometry and bioanalytical techniques. *Journal of Chromatography A*.

[71] Biau S, Bayle S, Barbara PD, Roig B (2007). The chick embryo: an animal model for detection of the effects of hormonal compounds. *Analytical and Bioanalytical Chemistry*.

[72] Isenhower WD, Newbold RR, Cefalo RC, Korach KS, McLachlan JA (1986). Absence of estrogenic activity in some drugs commonly used during pregnancy. *Biological Research in Pregnancy and Perinatology*.

[73] Padilla-Banks E, Jefferson WN, Newbold RR (2001). The immature mouse is a suitable model for detection of estrogenicity in the uterotropic bioassay. *Environmental Health Perspectives*.

[74] Kanno J, Onyon L, Haseman J, Fenner-Crisp P, Ashby J, Owens W (2001). The OECD program to validate the rat uterotrophic bioassay to screen compounds for *in vivo* estrogenic responses: phase 1. *Environmental Health Perspectives*.

[75] Flouriot G, Pakdel F, Ducouret B, Valotaire Y (1995). Influence of xenobiotics on rainbow trout liver estrogen receptor and vitellogenin gene expression. *Journal of Molecular Endocrinology*.

[76] Tyler CR, Sumpter JP, Witthames PR (1990). The dynamics of oocyte growth during vitellogenesis in the rainbow trout (*Oncorhynchus mykiss*). *Biology of Reproduction*.

[77] Knudsen FR, Schou AE, Wiborg ML (1997). Increase of plasma vitellogenin concentration in rainbow trout (*Oncorhynchus mykiss*) exposed to effluents from oil refinery treatment works and municipal sewage. *Bulletin of Environmental Contamination and Toxicology*.

[78] Brion F, Le Page Y, Piccini B (2012). Screening estrogenic activities of chemicals or mixtures *in vivo* using transgenic (cyp19a1b-GFP) zebrafish embryos. *PLoS ONE*.

[79] Ciana P, Di Luccio G, Belcredito S (2001). Engineering of a mouse for the *in vivo* profiling of estrogen receptor activity. *Molecular Endocrinology*.

[80] Lee O, Takesono A, Tada M, Tyler CR, Kudoh T (2012). Biosensor zebrafish provide new insights into potential health effects of environmental estrogens. *Environmental Health Perspectives*.

[81] Chen H, Hu J, Yang J (2010). Generation of a fluorescent transgenic zebrafish for detection of environmental estrogens. *Aquatic Toxicology*.

[82] Soto AM, Sonnenschein C, Chung KL, Fernandez MF, Olea N, Serrano FO (1995). The E-SCREEN assay as a tool to identify estrogens: an update on estrogenic environmental pollutants. *Environmental Health Perspectives*.

[83] Balaguer P, François F, Comunale F (1999). Reporter cell lines to study the estrogenic effects of xenoestrogens. *Science of the Total Environment*.

[84] Habauzit D, Boudot A, Kerdivel G, Flouriot G, Pakdel F (2010). Development and validation of a test for environmental estrogens: checking xeno-estrogen activity by CXCL12 secretion in Breast Cancer Cell Lines (CXCL-test). *Environmental Toxicology*.

[85] Swart JC, Pool EJ, van Wyk JH (2011). The implementation of a battery of *in vivo* and *in vitro* bioassays to assess river water for estrogenic endocrine disrupting chemicals. *Ecotoxicology and Environmental Safety*.

[86] Legler J, van den Brink CE, Brouwer A (1999). Development of a stably transfected estrogen receptor-mediated luciferase reporter gene assay in the human T47D breast cancer cell line. *Toxicological Sciences*.

[87] Arnold SF, Robinson MK, Notides AC, Guillette LJ, McLachan JA (1996). A yeast estrogen screen for examining the relative exposure of cells to natural and xenoestrogens. *Environmental Health Perspectives*.

[88] Petit F, Le Goff P, Cravédi JP, Valotaire Y, Pakdel F (1997). Two complementary bioassays for screening the estrogenic potency of xenobiotics: recombinant yeast for trout estrogen receptor and trout hepatocyte cultures. *Journal of Molecular Endocrinology*.

[89] Petit F, Valotaire Y, Pakdel F (1995). Differential functional activities of rainbow trout and human estrogen receptors expressed in the yeast *Saccharomyces cerevisiae*. *European Journal of Biochemistry*.

[90] Le Page Y, Scholze M, Kah O, Pakdel F (2006). Assessment of xenoestrogens using three distinct estrogen receptors and the zebrafish brain aromatase gene in a highly responsive glial cell system. *Environmental Health Perspectives*.

[91] Andersen HR, Andersson AM, Arnold SF (1999). Comparison of short-term estrogenicity tests for identification of hormone-disrupting chemicals. *Environmental Health Perspectives*.

[92] Fujimoto N, Honda H, Kitamura S (2004). Effects of environmental estrogenic chemicals on AP1 mediated transcription with estrogen receptors *α* and *β*. *Journal of Steroid Biochemistry and Molecular Biology*.

[93] Habauzit D, Flouriot G, Pakdel F, Saligaut C (2011). Effects of estrogens and endocrine-disrupting chemicals on cell differentiation-survival-proliferation in brain: contributions of neuronal cell lines. *Journal of Toxicology and Environmental Health B*.

[94] Shelby MD, Newbold RR, Tully DB, Chae K, Davis VL (1996). Assessing environmental chemicals for estrogenicity using a combination of *in vitro* and *in vivo* assays. *Environmental Health Perspectives*.

[95] Clark LC, Lyons C (1962). Electrode systems for continuous monitoring in cardiovascular surgery. *Annals of the New York Academy of Sciences*.

[96] Rodriguez-Mozaz S, Marco MP, de Alda MJL, Barceló D (2004). Biosensors for environmental monitoring of endocrine disruptors: a review article. *Analytical and Bioanalytical Chemistry*.

[97] D’Ursi P, Salvi E, Fossa P, Milanesi L, Rovida E (2005). Modelling the interaction of steroid receptors with endocrine disrupting chemicals. *BMC Bioinformatics*.

[98] Kanso H, Barthelmebs L, Inguimbert N, Noguer T (2013). Immunosensors for estradiol and ethinylestradiol based on new synthetic estrogen derivatives: application to wastewater analysis. *Analytical Chemistry*.

[99] Usami M, Mitsunaga K, Ohno Y (2002). Estrogen receptor binding assay of chemicals with a surface plasmon resonance biosensor. *Journal of Steroid Biochemistry and Molecular Biology*.

[100] Seifert M, Haindl S, Hock B (1999). Development of an enzyme linked receptor assay (ELRA) for estrogens and xenoestrogens. *Analytica Chimica Acta*.

[101] Rich RL, Hoth LR, Geoghegan KF (2002). Kinetic analysis of estrogen receptor/ligand interactions. *Proceedings of the National Academy of Sciences of the United States of America*.

[102] Fechner P, Pröll F, Carlquist M, Proll G (2009). An advanced biosensor for the prediction of estrogenic effects of endocrine-disrupting chemicals on the estrogen receptor alpha. *Analytical and Bioanalytical Chemistry*.

[103] Paulmurugan R, Gambhir SS (2006). An intramolecular folding sensor for imaging estrogen receptor-ligand interactions. *Proceedings of the National Academy of Sciences of the United States of America*.

[104] Kim SB, Sato M, Tao H (2009). Molecular tension-indexed bioluminescent probe for determining protein-protein interactions. *Bioconjugate Chemistry*.

[105] Sung BK, Umezawa Y, Kanno KA, Tao H (2008). An integrated-molecule-format multicolor probe for monitoring multiple activities of a bioactive small molecule. *ACS Chemical Biology*.

[106] Jisa E, Dornstauder E, Ogawa S, Inoue S, Muramatsu M, Jungbauer A (2001). Transcriptional activities of estrogen receptor alpha and beta in yeast properties of raloxifene. *Biochemical Pharmacology*.

[107] Berthier A, Elie-Caille C, Lesniewska E, Delage-Mourroux R, Boireau W (2011). Label-free sensing and atomic force spectroscopy for the characterization of protein-DNA and protein-protein interactions: application to estrogen receptors. *Journal of Molecular Recognition*.

[108] Cheskis BJ, Karathanasis S, Lyttle CR (1997). Estrogen receptor ligands modulate its interaction with DNA. *The Journal of Biological Chemistry*.

[109] Habauzit D, Armengaud J, Roig B, Chopineau J (2008). Determination of estrogen presence in water by SPR using estrogen receptor dimerization. *Analytical and Bioanalytical Chemistry*.

[110] Habauzit D, Chopineau J, Roig B (2007). SPR-based biosensors: a tool for biodetection of hormonal compounds. *Analytical and Bioanalytical Chemistry*.

[111] Peh WYX, Reimhult E, Huey FT, Thomsen JS, Su X (2007). Understanding ligand binding effects on the conformation of estrogen receptor *α*-DNA complexes: a combinational quartz crystal microbalance with dissipation and surface plasmon resonance study. *Biophysical Journal*.

[112] Bouter A, Buisine N, Le Grand A (2010). Control of vitellogenin genes expression by sequences derived from transposable elements in rainbow trout. *Biochimica et Biophysica Acta*.

[113] Szatkowski Ozers M, Hill JJ, Ervin K, Royer CA, Gorski J (2001). The dissociation rate of estrogen receptor *α* from the consensus estrogen response element. *Molecular and Cellular Endocrinology*.

[114] Ozers MS, Hill JJ, Ervin K (1997). Equilibrium binding of estrogen receptor with DNA using fluorescence anisotropy. *The Journal of Biological Chemistry*.

[115] Tamrazi A, Carlson KE, Daniels JR, Hurth KM, Katzenellenbogen JA (2002). Estrogen receptor dimerization: ligand binding regulates dimer affinity and dimer dissociation rate. *Molecular Endocrinology*.

[116] Swedenborg E, Pongratz I (2010). AhR and ARNT modulate ER signaling. *Toxicology*.

[117] Newbold RR, Hanson RB, Jefferson WN, Bullock BC, Haseman J, McLachlan JA (1998). Increased tumors but uncompromised fertility in the female descendants of mice exposed developmentally to diethylstilbestrol. *Carcinogenesis*.

[118] Jirtle RL, Skinner MK (2007). Environmental epigenomics and disease susceptibility. *Nature Reviews Genetics*.

[119] Anway MD, Cupp AS, Uzumcu N, Skinner MK (2005). Toxicology: epigenetic transgenerational actions of endocrine disruptors and male fertility. *Science*.

[120] Anway MD, Leathers C, Skinner MK (2006). Endocrine disruptor vinclozolin induced epigenetic transgenerational adult-onset disease. *Endocrinology*.

[121] Chang HS, Anway MD, Rekow SS, Skinner MK (2006). Transgenerational epigenetic imprinting of the male germline by endocrine disruptor exposure during gonadal sex determination. *Endocrinology*.

[122] Kavlock R, Cummings A (2005). Mode of action: inhibition of androgen receptor function—vinclozolin-induced malformations in reproductive development. *Critical Reviews in Toxicology*.

[123] Manikkam M, Guerrero-Bosagna C, Tracey R, Haque MM, Skinner MK (2012). Transgenerational actions of environmental compounds on reproductive disease and identification of epigenetic biomarkers of ancestral exposures. *PLoS ONE*.

[124] Molina-Molina JM, Hillenweck A, Jouanin I (2006). Steroid receptor profiling of vinclozolin and its primary metabolites. *Toxicology and Applied Pharmacology*.

[125] Nilsson E, Larsen G, Manikkam M, Guerrero-Bosagna C, Savenkova MI, Skinner MK (2012). Environmentally induced epigenetic transgenerational inheritance of ovarian disease. *PLoS ONE*.

[126] Revankar CM, Cimino DF, Sklar LA, Arterburn JB, Prossnitz ER (2005). A transmembrane intracellular estrogen receptor mediates rapid cell signaling. *Science*.

[127] Filardo EJ, Quinn JA, Frackelton AR, Bland KI (2002). Estrogen action via the G protein-coupled receptor, GPR30: stimulation of adenylyl cyclase and cAMP-mediated attenuation of the epidermal growth factor receptor-to-MAPK signaling axis. *Molecular Endocrinology*.

[128] Maggiolini M, Vivacqua A, Fasanella G (2004). The G protein-coupled receptor GPR30 mediates c-fos up-regulation by 17*β*-estradiol and phytoestrogens in breast cancer cells. *The Journal of Biological Chemistry*.

[129] Vivacqua A, Bonofiglio D, Albanito L (2006). 17*β*-estradiol, genistein, and 4-hydroxytamoxifen induce the proliferation of thyroid cancer cells through the G protein-coupled receptor GPR30. *Molecular Pharmacology*.

[130] Thomas P, Dong J (2006). Binding and activation of the seven-transmembrane estrogen receptor GPR30 by environmental estrogens: a potential novel mechanism of endocrine disruption. *Journal of Steroid Biochemistry and Molecular Biology*.

[131] Watson CS, Alyea RA, Jeng YJ, Kochukov MY (2007). Nongenomic actions of low concentration estrogens and xenoestrogens on multiple tissues. *Molecular and Cellular Endocrinology*.

[132] Tiefenbach J, Moll PR, Nelson MR (2010). A live zebrafish-based screening system for human nuclear receptor ligand and cofactor discovery. *PloS ONE*.

